# A cluster of autochthonous dengue transmission in the Paris region – detection, epidemiology and control measures, France, October 2023

**DOI:** 10.2807/1560-7917.ES.2023.28.49.2300641

**Published:** 2023-12-07

**Authors:** Nelly Fournet, Nathalie Voiry, Julian Rozenberg, Clément Bassi, Caroline Cassonnet, Anaïs Karch, Guillaume Durand, Gilda Grard, Gabriela Modenesi, Stevens-Boris Lakoussan, Nicolas Tayliam, Marta Zatta, Sébastien Gallien, on behalf of the investigation team, Harold Noël, Ségolène Brichler, Arnaud Tarantola

**Affiliations:** 1 Santé publique France (French National Public Health Agency), Saint-Denis, France; 2 Regional Health Agency of Île-de-France (ARS Île-de-France), Saint-Denis, France; 3 Regional Health Agency of Île-de-France (ARS Île-de-France), Créteil, France; 4 Agence régionale de Démoustication, Rosny-sous-Bois, France; 5 National Reference Center for Arboviruses, National Institute of Health and Medical Research (Inserm) and French Armed Forces Biomedical Research Institute (IRBA), Marseille, France; 6 Unité des Virus Émergents (UVE: Aix-Marseille Univ-IRD 190-Inserm 1207), Marseille, France; 7 Medical laboratory, Limeil-Brévannes, France; 8 Department of Infectious Diseases, Henri Mondor University Hospital, Assistance Publique-Hôpitaux de Paris, Paris, France; 9 The members of the investigation team are listed under Acknowledgements; 10 Santé publique France (French National Public Health Agency), Saint-Maurice, France; 11 Laboratory of virology, Avicenne University Hospital, Assistance Publique-Hôpitaux de Paris, Bobigny, France

**Keywords:** infectious disease outbreaks, dengue, autochthonous transmission, viral infections, France

## Abstract

A cluster of three confirmed autochthonous dengue cases was detected in October 2023 in the Val-de-Marne department neighbouring Paris, France. This marks the northernmost transmission of dengue in Europe reported to date. The epidemiological and microbiological investigations and the vector control measures are described. This event confirms the need for early case detection and response to contain dengue in Europe, especially given the 2024 Summer Olympic and Paralympic Games, when millions of visitors will visit the Greater Paris area.

Autochthonous transmission of dengue virus (DENV) has been limited to the southern regions of France until recently, with a marked increase in cases observed in 2022 [1]. In September 2023, the French arbovirus surveillance system detected the first autochthonous case of dengue in the Île-de-France region. Here, we report a cluster of three familial cases with autochthonous transmission of DENV in the Paris area of Île-de-France and describe the epidemiological and microbiological investigations, as well as the vector control measures.

## Detection of autochthonous cases

On 27 September 2023, a probable dengue case was identified through our surveillance system [1] and notified to the Regional Health Agency (ARS) in the Île-de-France region. A probable case is defined as a case with fever and detection of dengue-specific immunoglobulin M (IgM) antibodies in a single serum sample [2]. A blood sample taken from the patient on 22 September tested positive for anti-DENV IgM and negative for IgG, following discharge from a 2-day hospitalisation. The patient had presented fever and headache with an onset of symptoms on 13 September. A preserved blood sample collected on 21 September (8 days after symptom onset) during hospitalisation [3] was sent to the virology laboratory and confirmed positive on 10 October for DENV NS1 antigen with a weakly positive RT-PCR. The case had not travelled abroad or to southern France in the 15 days before symptom onset and had no associated morbidities or risk factors. The case resided in the Val-de-Marne department, near Paris, on the ground floor of a garden flat and had reported substantial mosquito nuisances and numerous mosquito bites.

Two family members of the index case presented similar symptoms on 11 and 14 September, neither of whom had travelled. Both were tested on 13 October at the same virology laboratory as the index, and samples returned positive for both anti-DENV IgM and IgG.

The only common place of exposure to mosquito bites during the incubation period for all three cases was their apartment.

## Epidemiological investigations

Upon detection of this cluster, the ARS and Santé publique France carried out active case finding to determine the extent of the dengue transmission, including (i) a press release on 17 October [4]; (ii) an email on 18 October to health professionals in the index case’s municipality informing them of the autochthonous cases, case definitions for testing and how to report patients with confirmed DENV infection to the health authorities, irrespective of travel history; and (iii) an active, door-to-door, case-finding survey on 19 and 20 October. The latter entailed visiting all households within a 150 m radius area around the index case’s home to identify any resident with sudden fever in the last 2 months without respiratory symptoms or who had recently travelled to a DENV-endemic area or southern France. Individuals reporting fever were asked to provide a fingertip blood spot sample.

While the survey was ongoing, flyers were distributed in residents’ mailboxes to inform them of local dengue transmission, but also to raise awareness about precautions to take in case of symptoms as well as the need to protect themselves from mosquito bites and limit the development of mosquito breeding sites, i.e. stagnant water. Additionally, a retrospective review of our laboratory surveillance data was carried out to identify any potentially imported primary dengue cases in the neighbourhood that could explain the local transmission.

Of the 305 households identified in the study area, 141 (46%) were visited, while in the other 164 households, inhabitants were not home at the time of the survey; 16 individuals were identified who reported a fever in the past 2 months. Fingertip blood spots were collected on filter paper for 10 individuals, and six declined sampling. All were handed prescriptions for serology testing and letters for their general practitioner. We did not identify any individual with a recent travel history to an area with active dengue circulation that could correspond to a primary case as a source of local transmission.

## Laboratory investigations

On 16 October, the National Reference Centre for arboviruses (NRC) in Marseille, France, confirmed the presence of DENV-2 RNA using an in-house PCR [5] and the presence of anti-DENV IgM in the sample collected on 21 September from the index case. The viral load was too low to sequence the virus because sample collection occurred 8 days after symptom onset. The NRC also confirmed positive serology for anti-DENV IgM and IgG for the two family members.

On 30 October, the NRC returned negative results for IgM and IgG of fingertip blood spot analysis from the 10 individuals sampled during the epidemiological investigations. In addition, three of these 10 suspected cases underwent laboratory testing, along with one of the six other suspected cases who declined the blood spot analysis. Three other individuals living in the municipality were found in our laboratory surveillance data as having been tested after the door-to-door case-finding survey. All 7 individuals tested negative for DENV serology or PCR.

## Entomological investigations and vector control measures

Following the positive result of DENV infection for the index case, the Agence Régionale de Démoustication (ARD), responsible for vector control, initiated entomological investigations on 12 October on the grounds and within a 150 m radius around the index case's residence. The investigations showed substantial presence of adult *Aedes albopictus* mosquitoes. The ARD distributed flyers to inform residents of local dengue transmission, of the upcoming mosquito control operation and of the prevention measures addressing mosquito larvae in breeding sites. An adulticide treatment using deltamethrin was applied to decrease the risk of transmission on the resting areas of mosquitoes (vegetation) during the night of Sunday 15 October, and a second treatment occurred 7 days later.

## Arbovirus surveillance in the Île-de-France region

Dengue is a mandatory notifiable disease year-round in mainland France. Surveillance of human cases is enhanced during *Ae. albopictus*‘s estimated activity period (from May to November) [6] by Santé publique France regional teams, with a daily review of arbovirus diagnostic tests conducted in a network of laboratories to identify cases not notified through the mandatory notification system [1].

**Figure 1:**
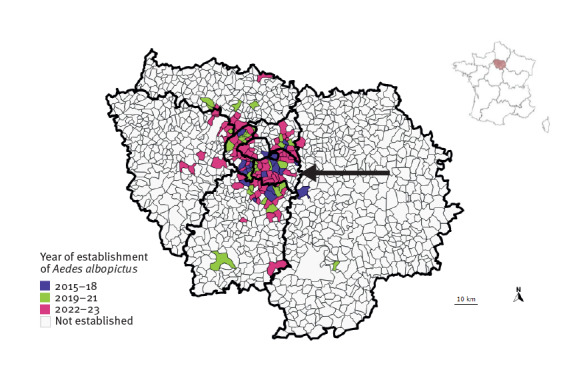
Spatial distribution and year of colonisation of *Aedes albopictus*, Île-de-France region, France, 2015–2023

**Figure 2:**
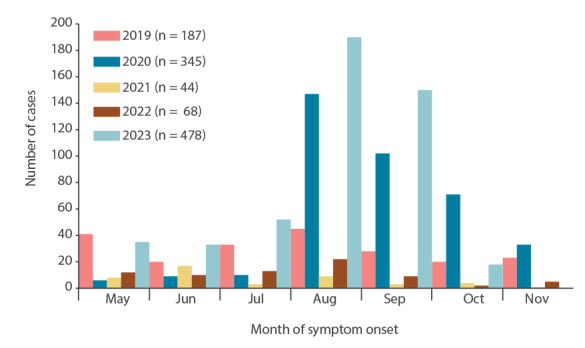
Distribution of imported dengue cases by month of symptom onset, during the enhanced surveillance between May and November, Île-de-France region, France, 2019 up to 13 October 2023

The *Ae. albopictus* mosquito has been present in the Île-de-France region (comprised of seven departments including Paris) since 2015, when it was first detected in the Val-de-Marne department. *Aedes albopictus* is now considered established and active in all departments of Île-de-France (Figure 1).The Île-de-France region accounts for the highest proportion of imported dengue cases annually in mainland France, ranging from 28% to 41% between 2019 and 2023. In 2023, we observed an increased number of imported cases, with 478 cases up to 13 October (Figure 2), including 70% from Guadeloupe and Martinique where a dengue epidemic was ongoing [7].

Source of data: national surveillance system of invasive mosquito, SI-LAV, Ministry of health. The last update of data was on 15 September 2023. The Val-de-Marne department is indicated by the black arrow.

The map was generated with the Magrit mapping application (http://magrit.cnrs.fr).

Source of data: French national arbovirus surveillance system, Santé publique France.

## Discussion

This intra-familial cluster of three autochthonous cases of dengue is, to our knowledge, the first identified in the Île-de-France region and the northernmost transmission of dengue in Europe reported up to 30 November 2023. This occurrence may represent an epidemiological marker in dengue transmission in Europe. Previously, autochthonous transmissions of DENV as well as chikungunya and zika viruses had only been described in the southern regions of France [8-12]. A marked increase in dengue was observed in 2022 [1], while other European countries bordering the Mediterranean have also reported episodes of local transmission of DENV [13,14].

With two international airports and being the most densely populated region in France (12.4 million inhabitants as on 1 January 2023 [15]), Île-de-France experiences important population movements to and from dengue-endemic areas. The rapidly growing number of municipalities colonised by *Ae. albopictus* consequently increases the proportion of the population exposed to the risk of local transmission. The case’s municipality of residence was not officially considered colonised by *Ae. albopictus*, based on the 2022 entomological data. Given the combined influence of climate change – with 2023 registering the highest September temperatures in France since 1900 [16,17] – and other environmental factors, in particular in densely vegetated areas similar to the one where the transmission occurred, the risk of autochthonous transmission is well-established and increasing [18-20]. In addition, *Aedes* mosquitoes continually extend their range into northern Europe [21]. Of more immediate concern, millions of visitors from around the world, including those from endemic areas, will visit the region next July to September for the Paris 2024 Summer Olympic and Paralympic Games. It is essential to mitigate the risk of local transmission posed by vector proliferation and to enhance case detection by shortening the diagnosis and notification delay.

Delay in diagnosis and notification is a major risk factor for autochthonous transmission [22], as it could prolong control measures. The diagnosis of this autochthonous dengue cluster was based on the initiatives of the medical laboratory professionals who recorded an imported dengue case a few weeks earlier in the same municipality, and of the hospital physicians, following the lack of a diagnosis for febrile acute hepatitis [3]. The door-to-door investigation did not identify any other cases or an index case that could have initiated the transmission.

Our investigation had some limitations. Firstly, given the low volume of sera, and the high cycle threshold in the PCR, the virus isolation was not attempted for the index case. Genomic analyses are important to characterise strains and are performed routinely by the NRC for imported and autochthonous cases. Unfortunately, these analyses could not be performed because there were no samples collected within 7 days of symptom onset available for the three cases, and only serology could be implemented for the last two cases. Secondly, negative results on fingertip blood spot tests do not entirely rule out infection given the suspected lower sensitivity of the test compared with a venous blood sample analysis and only few cases provided samples for laboratory serology testing, which were all negative. Thirdly, nearly half of the households in the affected neighbourhood could not be visited during our door-to-door survey. Finally, asymptomatic infections could have gone undetected by the surveillance system or by our survey. However, given the information widely distributed through flyers in mailboxes and on the doors of buildings, and with local healthcare professionals sensitised about dengue diagnosis and prevention along with media attention, it seems unlikely that a substantial number of symptomatic cases would have gone unnoticed.

## Conclusion

This episode of local dengue transmission in the Paris region highlights that cases of arboviral diseases have begun to emerge in northern France and could emerge in the near future in the rest of Europe. Preparedness should begin to shift towards adapting the monitoring and response efforts to a larger scale. Actions must be taken in several areas, including the implementation of prevention and control measures by both the population and municipalities to reduce larval breeding sites by eliminating all stagnant water. In addition, raising awareness among healthcare professionals to consider chikungunya, dengue and zika as diagnoses in the presence of fever without other infectious causes and among the population to seek medical attention upon returning from an endemic area and experiencing fever is of importance.
